# Profiling of Metabolome in the Plasma Following a circH19 Knockdown Intervention in Diet-Induced Obese Mice

**DOI:** 10.3390/metabo14110603

**Published:** 2024-11-08

**Authors:** Hanxin Zhao, Dike Shi, Weiwei Gui, Xihua Lin, Jionghuang Chen, Weihua Yu

**Affiliations:** 1 Department of Endocrinology, Sir Run Run Shaw Hospital, School of Medicine, Zhejiang University, Hangzhou 310016, China; z63hanxin@zju.edu.cn (H.Z.); guiweiwei@zju.edu.cn (W.G.); linxihua@zju.edu.cn (X.L.); 2 Department of Gastroenterology Surgery, The Second Affiliated Hospital, School of Medicine, Zhejiang University, Hangzhou 310016, China; 2314044@zju.edu.cn; 3 Department of General Surgery, Sir Run Run Shaw Hospital, School of Medicine, Zhejiang University, Hangzhou 310016, China

**Keywords:** CircRNA H19, obesity, metabolites, liquid chromatography–mass spectrometry

## Abstract

The circular RNA circH19 has been implicated in the regulation of gene expression and various biological processes, including obesity. **Objectives**: This study aimed to elucidate the metabolic changes in plasma after circH19 knockdown in a diet-induced obese (DIO) mouse model. **Methods:** Plasma samples were collected following the intervention and subjected to non-targeted metabolomics analysis using liquid chromatography–mass spectrometry (LC-MS). Metabolic profiling was performed to identify and quantify metabolites, followed by multivariate statistical analysis to discern differential metabolic signatures. **Results:** A total of 1250 features were quantified, resulting in the upregulation of 564 metabolites and the downregulation of 686 metabolites in the circH19 knockdown group compared to the control mice. Metabolic pathway analysis revealed disruptions in lipid metabolism, amino acid turnover, and energy production pathways. Notably, the intervention led to a substantial decrease in circulating lipids and alterations in the plasma amino acid profile, indicative of an impact on protein catabolism and anabolic processes. The observed shifts in lipid and amino acid metabolism suggest potential therapeutic avenues for obesity and related metabolic disorders. **Conclusions:** The circH19 knockdown in DIO mice led to significant alterations in plasma metabolites, highlighting its potential role in the regulation of obesity and metabolic disorders.

## 1. Introduction

Obesity has reached epidemic proportions globally, posing a significant threat to public health. It is widely recognized as a multifactorial disorder influenced by genetic, environmental, and behavioral factors that lead to an excessive accumulation of body fat [1]. The molecular underpinnings of obesity are complex, involving intricate regulatory networks that govern energy homeostasis, adipogenesis, and lipid metabolism [2,3]. With the advent of systems biology, there has been a shift towards understanding obesity at the systemic level, emphasizing the need to explore the metabolome—the full repertoire of small molecules within a biological system [4,5].

Circular RNAs (circRNAs), a class of non-coding RNAs, have emerged as key regulators of gene expression and function. They are characterized by their covalently closed loop structure, which confers stability and resistance to degradation by exonucleases [6]. CircRNAs are increasingly recognized for their roles in metabolic processes through various molecular mechanisms, including acting as miRNA sponges to regulate the availability of miRNAs, thereby affecting the expression of target genes involved in metabolism. CircRNAs can interact with RNA-binding proteins, influencing their activity or subcellular localization, which in turn modulates metabolic pathways by altering the function or stability of these proteins. Moreover, circRNAs have been shown to participate in the epigenetic regulation of gene expression, potentially by guiding chromatin modifiers to specific genomic loci, thus impacting the transcription of genes crucial for metabolic homeostasis [6,7]. These pathways underscore the complex regulatory network within which circRNAs operate to influence metabolic health and disease.

Has_circH19 derived from long non-coding RNA (lncRNA) H19 pre-RNA, which is also called hsa_circ_0095570 in circBase, is located in chr11:2018820–2019000, with H19 as the gene symbol [8]. H19 has been demonstrated to exert a crucial influence on lipid metabolism. Elevated levels of H19 have been shown to confer protection against obesity and enhance insulin sensitivity [9]. CircH19, in particular, has been implicated in various biological processes and diseases, including cancer and metabolic disorders [10]. The role of circH19 in obesity, however, remains largely unexplored.

Currently, the emergence of metabolic disorders, including obesity and type 2 diabetes mellitus, is associated with perturbations across various interconnected ‘omic’ dimensions encompassing the transcriptomic, epigenomic, and metabolomic levels. Such multi-layered ‘omic’ analyses contribute to deciphering the complex physiological mechanisms at play in these conditions [11,12]. The plasma metabolome represents a dynamic reflection of an individual’s metabolic status, influenced by both endogenous and exogenous factors. It serves as a valuable source of biomarkers for the diagnosis and monitoring of metabolic disorders, including obesity [13]. Non-targeted metabolomics, an unbiased analytical approach, allows for the comprehensive profiling of metabolites in biological samples. This method has been increasingly applied to studying the metabolic consequences of obesity and related comorbidities [2].

In this study, we aimed to investigate the metabolic changes in the plasma of DIO mice following a circH19 knockdown intervention. We hypothesized that modulating circH19 levels would result in discernible metabolic alterations that could provide insights into the role of circH19 in obesity pathophysiology. By employing non-targeted metabolomics, we sought to identify specific metabolic pathways and processes affected by circH19 knockdown, thus potentially uncovering novel mechanisms and therapeutic targets for obesity.

## 2. Materials and Methods

### 2.1. Animals and Animal Treatments

The guidelines of the Animal Care Committee of Zhejiang University were strictly followed in all protocols involving animals. The study mice, male C57BL/6 aged 8 weeks, were commercially obtained from the Slack Experimental Animal Center of the Chinese Academy of Sciences (Shanghai, China). The mice were housed in a specific pathogen-free environment and maintained on a 12 h light–dark cycle. High-fat diet (HFD)-induced obesity was established by feeding the mice a diet consisting of 45% fat, 20% protein, and 35% carbohydrates (Research Diets, D12451) for 12 weeks. Throughout this period, the mice had libitum access to food and water and were maintained under controlled environmental conditions.

The HFD group was further divided into two treatment groups after the development of obesity following 12 weeks on the high-fat diet. The mice in each group were administered their respective adenoviruses via tail vein injection at a concentration of 1 × 10^11^ virus genomic particles per milliliter twice weekly for 2 weeks. This intervention was intended to knockdown circH19 specifically in the treatment group. Six were treated with control adenovirus, and six were treated with circH19 knockdown adenovirus. After 16 weeks, the mice were humanely sacrificed following a blood glucose level test, blood collection, and recording of body weights after an overnight fast for 12 h, which involved fasting without restriction of water intake. Blood glucose levels were measured from tail blood in the morning using a One Touch Ultra glucose meter and strips (LifeScan, Malvern, PA, USA). Blood samples were collected via cardiac puncture. These samples were immediately placed into tubes containing EDTA as an anticoagulant and centrifuged to separate the plasma. The plasma was then aliquoted and stored at −80 °C until further analysis for metabolomic profiling.

Subsequently, the weights of liver and inguinal white adipose tissues (ingWAT) were recorded, and the tissues were then subjected to fixation using a 4% formaldehyde solution (Sigma-Aldrich, St. Louis, MO, USA) for a duration of 24 h at room temperature, followed by dehydration in a graded ethanol series. Dehydration steps included an initial rinse in 70% ethanol (Fisher Scientific, Waltham, MA, USA) for 1 h, followed by 95% ethanol (Fisher Scientific, Waltham, MA, USA) for 1 additional hour, and finally two changes of 100% ethanol (Fisher Scientific, Waltham, MA, USA) for 30 min each. The OCT compound (Sakura Finetek, Torrance, CA, USA) was used to embed the dehydrated tissue samples, and sectioning was carried out using a Leica CM1950 cryotome with sections cut at a thickness of 4 μm intervals. Hematoxylin–eosin (H&E) staining was performed according to the standard protocol. The staining reagents were obtained from Thermo Fisher Scientific (Hematoxylin Solution, RAL555S, and Eosin Y Solution, RAL600S). Imaging was conducted using a Nikon Eclipse Ni-U microscope equipped with a Nikon DS-Ri2 camera. For the quantification of adipocyte size area measurements, the ImageJ software (National Institutes of Health, Bethesda, MD, USA) was utilized.

### 2.2. Adenovirus Knockdown

For recombinant adenovirus sh-circH19 construction, oligos with the circH19 target sequences were employed to clone shRNA-coding sequences into the pDC311-U6-MCMV-EGFP vector (Hanbio, Shanghai, China). The annealed oligos were directionally propagated into the BamH1/EcoRI-digested pDC311-U6-MCMV-EGFP vector. The pDC311-sh-circH19 with pBHGlox_E1, 3Cre [14] was co-inserted into HEK293 cells via transfection with the LipoFiterTM liposome transfection reagent (Hanbio, Shanghai, China) to create the recombinant adenoviruses (si-CircH19). Adenoviruses containing green fluorescent protein (si-Control) were employed as the control. Then, si-CircH19 and si-Control were inoculated into HEK293 cells. The cloned recombinant adenoviruses in the HEK293 cells were purified, and the titer of the virus was assessed through plaque assays. The stock solutions of sh-circH19 and sh-control were made with 1 × 10^11^ plaque formation unit (PFU)/mL.

### 2.3. Biochemical Indicator Test

All of the biochemical indicators were conducted after an overnight fast of the mice. The levels of triglycerides (TG), total cholesterol (TC), high-density lipoprotein cholesterol (HDL-c), and low-density lipoprotein cholesterol (LDL-c) were measured using a TG assay kit (A110-1-1), a TC assay kit (A111-1-1), an HDL-c assay kit (A112-1-1), and an LDL assay kit (A113-1-1) from Nanjing Jiancheng Bioengineering Institute, China. The measurement of free fatty acid (FFA) was conducted utilizing a Free Fatty Acid Fluorometric Assay Kit (Cayman Chemical, 70031096) according to the guidelines outlined by the manufacturer. Alanine aminotransferase (ALT), aspartate aminotransferase (AST), and alkaline phosphatase (ALP) were measured using an ALT assay kit (C009-2-1), an AST assay kit (C010-2-1), and an ALP assay kit (A059-2-2) from Nanjing Jiancheng Bioengineering Institute, China. The measurement procedure followed the instructions provided by the manufacturer.

Taking the specific steps of the TG detection Kit as an example, begin with the preparation of the serum samples for direct testing, and dilute the serum samples with physiological saline to ensure accurate measurements. Next, following the operation table instructions, dispense 2.5 μL of the diluted serum sample into the designated sample wells of a 96-well microplate. In the same manner, add 2.5 μL of distilled water to the blank wells and 2.5 μL of the assay kit’s standard to the standard wells. Then, add 250 μL of the working solution containing the necessary enzymes and substrates to each well. Afterward, gently mix the contents of the plate to ensure homogeneity and incubate the plate at 37 °C for 10 min to allow the enzymatic reaction to proceed. Once the incubation period is complete, use a microplate reader to measure the absorbance at a wavelength of 500 nm. The resulting data, which include the absorbance values of the blank, standard, and sample wells, are then used to calculate the triglyceride concentration in the mouse serum samples according to the provided calculation formula, taking into account the standard curve generated from the standard wells. This process yields a precise measurement of triglyceride levels in the serum to be further analyzed.

### 2.4. RNA Extraction and qRT-PCR Assay

Total RNA was isolated from samples using Trizol reagent (Invitrogen) following the manufacturer’s protocol. Then, the extracted total RNA was quantified using a NanoDrop 2000 (Thermo Fisher, Waltham, MA, USA). Then, 5 μg of RNA was treated with 40 units of RNase R (Geneseed, Guangdong, China) in the presence of 1 × RNase R buffer and incubated for 15 min at 37 °C and 10 min at 70 °C. Reverse transcription (RT) was performed using a reverse transcription reagents kit (TaKaRa, Otsu, Shiga, Japan) to generate a cDNA template following the manufacturer’s protocol. Real-time quantitative PCR was performed using a PCR kit containing SYBR Green (TaKaRa, Otsu, Shiga, Japan) on a 7500 real-time PCR detection system (Applied Biosystems, Carlsbad, CA, USA). The relative mRNA expression levels were calculated using the 2^−ΔΔCt^ method and normalized to *Gapdh* mRNA. The primer sets used are listed in Supplementary Table S1.

### 2.5. Metabolite Extraction

The collected samples were thawed on ice, and the metabolite were extracted with 80% methanol Buffer. Briefly, 100 μL of the sample was extracted with 400 μL of precooled methanol. The extraction mixture was then stored in 30 min at −20 °C. After centrifugation at 20,000× *g* for 15 min, the supernatants were transferred into a new tube and vacuum-dried. The samples were redissolved with 100 μL of 80% methanol and stored at −80 °C prior to the LC-MS analysis. In addition, pooled QC samples were also prepared by combining 10 μL of each extraction mixture.

### 2.6. Non-Targeted Metabolomics Analysis

All samples were acquired using the LC-MS system following machine orders. Firstly, all chromatographic separations were performed using an UltiMate 3000 UPLC System (Thermo Fisher Scientific, Bremen, Germany). An ACQUITY UPLC T3 column (100 mm × 2.1 mm, 1.8 µm, Waters, Milford, MA, USA) was used for the reversed phase separation. The column oven was maintained at 40 °C. After establishing the baseline conditions, a mobile phase comprising 5 mM ammonium acetate and 5 mM acetic acid (solvent A) was used in conjunction with Acetonitrile (solvent B) for chromatographic separation. The system operated at a flow rate of 0.3 mL/min to achieve optimal elution and resolution of analytes. Gradient elution conditions were set as follows: 0~0.8 min, 2% B; 0.8~2.8 min, 2% to 70% B; 2.8~5.6 min, 70% to 90% B; 5.6~6.4 min, 90% to 100% B; 6.4~8.0 min, 100% B; 8.0~8.1 min, 100% to 2% B; 8.1~10 min, 2% B.

A high-resolution tandem mass spectrometer Q-Exactive (Thermo Fisher Scientific, Bremen, Germany) was used to detect metabolites eluted from the column. The Q-Exactive was operated in both positive and negative ion modes. Precursor spectra (70–1050 *m*/*z*) were collected at 70,000 resolutions to hit an AGC target of 3 × 10^6^. The maximum inject time was set to 100 ms. A top 3 configuration to acquire data was set in DDA mode. Fragment spectra were collected at 17,500 resolutions to hit an AGC target of 1 × 10^5^ with a maximum inject time of 80 ms. In order to evaluate the stability of the LC-MS during acquisition, a quality control sample (pool of all samples) was acquired after every 10 samples.

### 2.7. Data Processing and Metabolite Identification

The acquired MS data pretreatments, including peak picking, peak grouping, retention time correction, second peak grouping, and annotation of isotopes and adducts, were performed using XCMS software. LC–MS raw data files were converted into mzXML format and then processed using the XCMS, CAMERA, and metaX toolbox implemented with R software. Each ion was identified by combining the retention time (RT) and *m*/*z* data. Intensities of each peak were recorded, and a three-dimensional matrix containing arbitrarily assigned peak indices (retention time–*m*/*z* pairs), sample names (observations), and ion intensity information (variables) was generated. The online KEGG, HMDB database was used to annotate the metabolites by matching the exact molecular mass data (*m*/*z*) of samples with those from database. If the mass difference between the observed and the database value was less than 10 ppm, the metabolite was annotated, and the molecular formula of the metabolites was further identified and validated using the isotopic distribution measurements.

We also used an in-house fragment spectrum library of metabolites to validate the metabolite identification. The intensity of the peak data was further preprocessed using metaX. Those features that were detected in less than 50% of QC samples or 80% of biological samples were removed, and the remaining peaks with missing values were imputed with the k-nearest neighbor algorithm to further improve the data quality. PCA was performed for outlier detection and batch effects evaluation using the pre-processed dataset. Quality-control-based robust LOESS signal correction was fitted to the QC data with respect to the order of injection to minimize signal intensity drift over time. We utilized univariate analysis of fold-change and *t*-test statistical tests with Benjamini–Hochberg (BH) correction to obtain q-values. Combined with multivariate statistical analysis using Partial Least Squares Discriminant Analysis (PLS-DA), the VIP (Variable Important for the Projection) values were obtained to screen for differentially expressed metabolic ions. The differential ions met the following criteria: ratio of ≥1.5 or ≤−1/1.5; *p*-value of <0.05; VIP value of ≥1.

### 2.8. Statistical Analysis

The relative standard deviations of the metabolic features were calculated across all QC samples, and those > 30% were then removed. Student *t*-tests were conducted to detect differences in metabolite concentrations between 2 phenotypes. The *p* value was adjusted for multiple tests using an FDR (Benjamini–Hochberg). Supervised PLS-DA was conducted through metaX to determine the different variables between groups. The VIP value was calculated. A VIP cut-off value of 1.0 was used to select important features. For the evaluation of dietary and treatment effects in the mouse study, we utilized one-way ANOVA followed by Tukey’s multiple comparison test to assess the significance of differences among the groups.

## 3. Results

### 3.1. CircH19 Knockdown Can Alleviate Lipid Metabolism Disorder in Obese Mice

**Figure 1:**
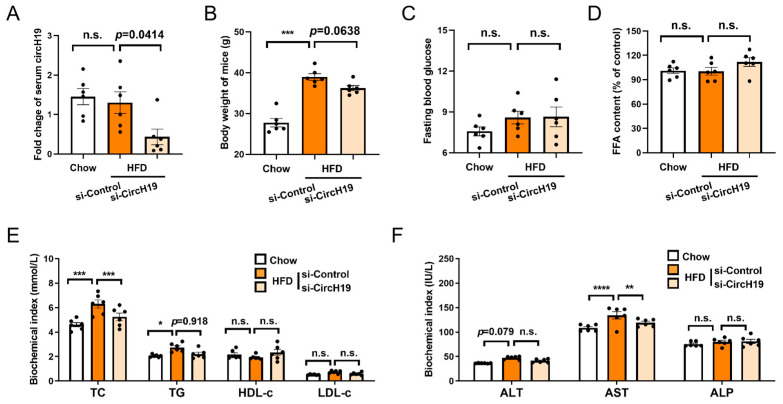
CircH19 knockdown impacts body weight and lipid metabolism in obese mice. (**A**) The mean serum levels of circH19 in Chow mice and HFD obese mice following treatment with si-Control or si-CircH19 as measured through qRT-PCR. Error bars represent the standard error of the mean (SEM). (**B**) The body weight of Chow mice and HFD obese mice after the treatment with si-Control or si-CircH19. (**C**) The fasting blood glucose of Chow mice and HFD obese mice after the treatment with si-Control or si-CircH19. (**D**) The serum FFA levels in Chow mice and HFD obese mice following circH19 knockdown. (**E**) The serum levels of total cholesterol (TC), triglycerides (TGs), high-density lipoprotein cholesterol (HDL-c), and low-density lipoprotein cholesterol (LDL-c) in Chow mice and HFD obese mice following circH19 knockdown. (**F**) The serum levels of ALT, AST, and ALP in Chow mice and HFD obese mice following circH19 knockdown. All n = 6, *p* < 0.05 indicates statistical significance analyzed through one-way ANOVA with a Tukey’s multiple comparisons test between HFD obese mice after the treatment with si-Control and si-CircH19. ‘n.s.’ (not significant), * (*p* < 0.05), ** (*p* < 0.01), *** (*p* < 0.001), **** (*p* < 0.0001).

After successfully inducing obesity in mice with a high-fat diet, to assess the efficacy of circH19 knockdown, serum samples were collected following the HFD obese mice that were treated with either si-Control or si-CircH19. Total circH19 levels in the serum were quantified using a qRT-PCR assay. The results revealed a significant decrease in circH19 levels in the serum of mice treated with si-CircH19 compared to those treated with si-Control, with *p* = 0.0426 (Figure 1A), confirming the successful knockdown of circH19. The circH19 knockdown intervention in obese mice led to a significant reduction in body weight (Figure 1B) but no significant change in fasting blood glucose levels (Figure 1C), indicating that circH19’s impact on obesity may not be directly associated with alterations in glucose homeostasis.

Next, we conducted tests on the lipid metabolic indicators total cholesterol (TC), triglycerides (TGs), free fatty acids (FFAs), high-density lipoprotein cholesterol (HDL-c), and low-density lipoprotein cholesterol (LDL-c) and the liver enzymes ALT, AST, and ALP in HFD obese mice (Figure 1E,F). After knockdown of circH19 in obese mice, we observed a significant reduction in the levels of TC and TG, as well as the liver enzyme AST, while the levels of HDL-c and FFA remained unchanged, suggesting a specific modulation of circH19 on lipid metabolism in obesity.

**Figure 2:**
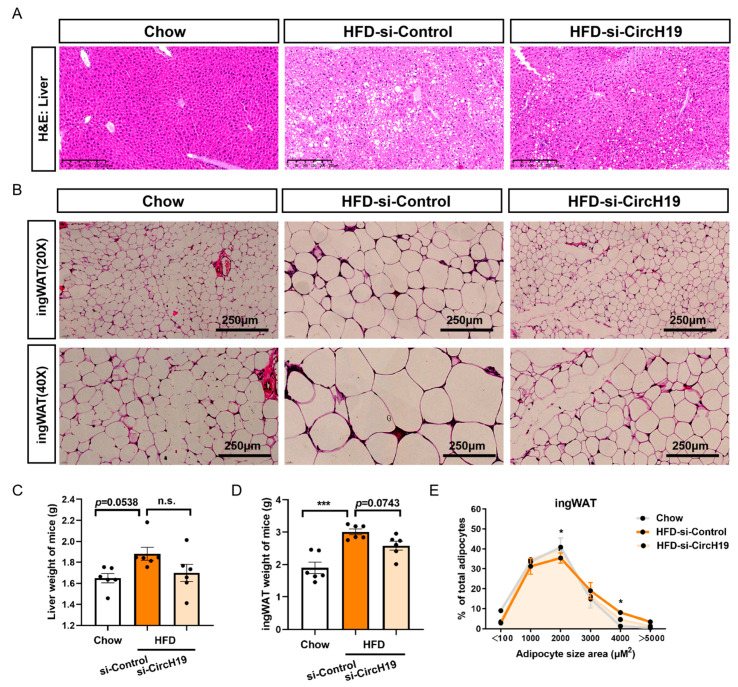
CircH19 knockdown alleviates lipid deposition in HFD-induced obese mice. (**A**) Hematoxylin/eosin representative staining of liver tissue in Chow mice and HFD obese mice following circH19 knockdown. Magnification: 20×. (**B**) Hematoxylin/eosin representative staining of adipocytes in inguinal white adipose tissue (ingWAT) in Chow mice and HFD obese mice following circH19 knockdown. Magnification: top panel, 20×; bottom panel, 40×. (**C**) The liver weight of Chow mice and HFD obese mice after the treatment with si-Control or si-CircH19. (**D**) The ingWAT weight of Chow mice and HFD obese mice after the treatment with si-Control or si-CircH19. (**E**) Adipocyte size distribution curves of ingWAT in Chow mice and HFD obese mice following circH19 knockdown. n = 6, * *p* < 0.05, analyzed through one-way ANOVA with a Tukey’s multiple comparisons test between HFD obese mice after the treatment with si-Control and si-CircH19. ‘n.s.’ (not significant), * (*p* < 0.05), *** (*p* < 0.001).

At the same time, hematoxylin/eosin staining of liver and inguinal white adipose tissue (ingWAT) revealed that circH19 knockdown alleviates liver lipid deposition and adipocyte enlargement (Figure 2A,B). Because the body weight of the HFD obese mice following circH19 knockdown was reduced, we measured the weight of the liver tissues and ingWAT in mice, and although improvement in lipid deposition was observed in the liver, there was no difference in the liver weight between HFD obese mice after the treatment with si-Control and si-CircH19 (Figure 2C). There was a significant decrease of ingWAT weight in the si-CircH19 group (Figure 2D). Adipocyte size distribution curves showed decreased adipocyte size in ingWAT between HFD obese mice after the treatment with si-Control and si-CircH19 (Figure 2E). In conclusion, the findings suggest that CircH19 knockdown can alleviate lipid deposition in HFD-induced obese mice.

### 3.2. Metabolite Detection Quality Control and Metabolite Identification and Quantification

**Figure 3:**
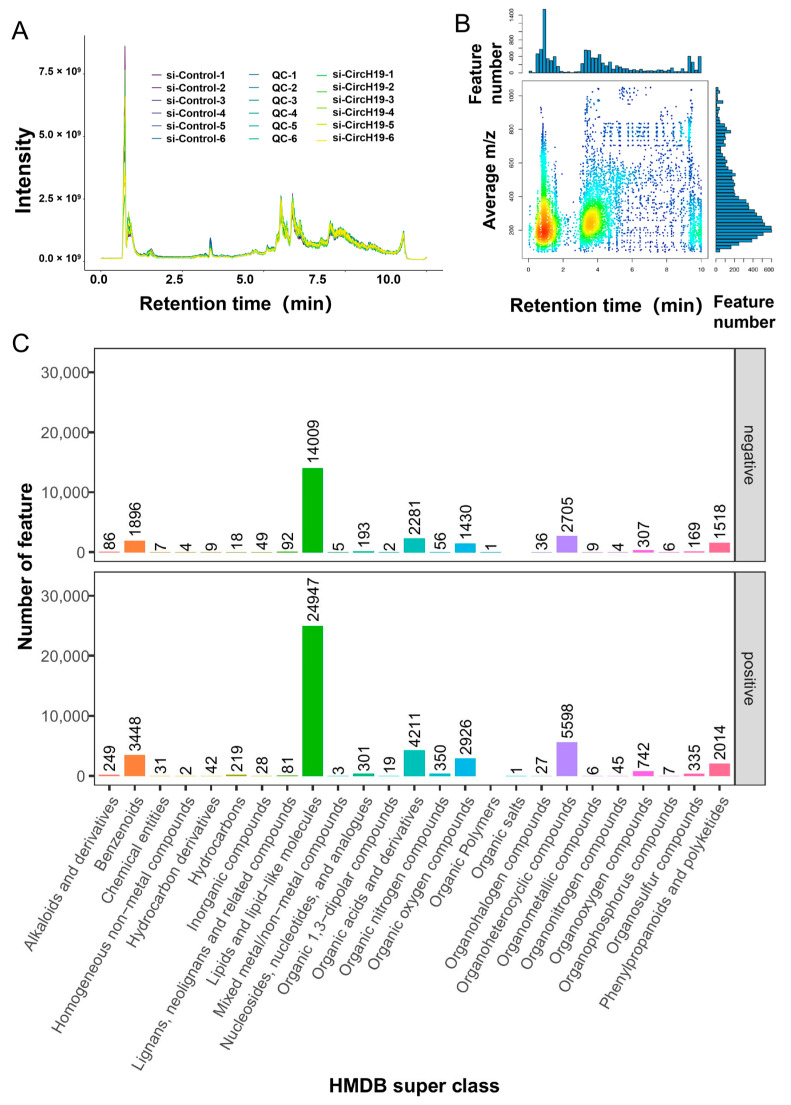
Metabolite extraction and LC-MS analysis. (**A**) Total ion chromatogram (TIC): The overall mass spectrum signal intensity of the controlled sample. The TIC diagram takes the time point as the horizontal coordinate and the total strength of all ions in the mass spectrum at each time point as the vertical coordinate, and each color represents a sample. TIC can macroscopically reflect the separation of all metabolites in liquid chromatography. (**B**) High-resolution mass spectrometers were used to detect different substances (see the description of experimental methods for specific types of mass spectrometers). In multi-sample detection, the *m*/*z* (±0.01) and retention time (±0.5 min) of the sample will change slightly. In this analysis, XCMS software was used to perform peak alignment processing on the mass spectrum data to quantify the metabolites more accurately. The graph takes the retention time as the horizontal coordinate and *m*/*z* as the vertical coordinate. Each point represents a substance, and the color indicates how dense the substance is in that area. The darker the color, the larger the number of feature numbers. (**C**) This histogram shows the distribution of metabolite features classified based on their superclass in the Human Metabolome Database (HMDB), detected in both positive and negative ionization modes.

Subsequently, serum samples from mice treated with si-CircH19 knockdown and from the control group were collected for untargeted metabolomics analysis. The total ion chromatogram (TIC) is a standard representation in untargeted metabolomics studies, showing the ion intensity as a function of retention time for a specific sample. In this chromatogram, the retention time scale ranges from 0 to 10.0 min, and the ion intensity scale reaches up to 7.5 × 10^9^ arbitrary units. The chromatographic peaks vary in height, corresponding to different metabolite species present in the sample, with the highest peak reaching approximately 5.0 × 10^9^ units. The provided data suggest that the sample contains a complex mixture of metabolites that have been separated based on their chemical properties during the chromatographic run (Figure 3A). The feature map visualizes the distribution of detected metabolites across different retention times and *m*/*z* ratios in an untargeted metabolomics analysis. This type of visualization shows the complexity of the metabolome in the sample, as it allows for the identification of metabolites based on their retention time and *m*/*z*, which are key parameters for metabolite characterization and identification (Figure 3B). The resulting chromatogram is a snapshot of the metabolite composition of the sample at a given point in time, providing a comprehensive overview of the metabolome.

A classification histogram of metabolites was identified in this study, categorized by their superclass in the Human Metabolome Database (HMDB). The x-axis represents the superclass of metabolites, while the y-axis indicates the count of metabolite features within each superclass. The bars are divided into two sections, one for positive mode detection and the other for negative mode detection, reflecting the ionization mode used during mass spectrometry analysis. The chart provides an overview of the metabolite diversity and relative abundance within each superclass, highlighting the most prevalent metabolite groups in the analyzed samples. The green bars specifically indicate the count of metabolite features classified within a particular superclass—lipid and lipid-like molecules—as identified by the HMDB (Figure 3C). The enrichment of lipids and lipid-like molecules in the metabolite profile suggests that knockdown of circH19 may improve the obese state in mice by affecting lipid metabolism.

### 3.3. Effect of circH19 Knockdown on Metabolic Profiles in Obese Mice

**Figure 4:**
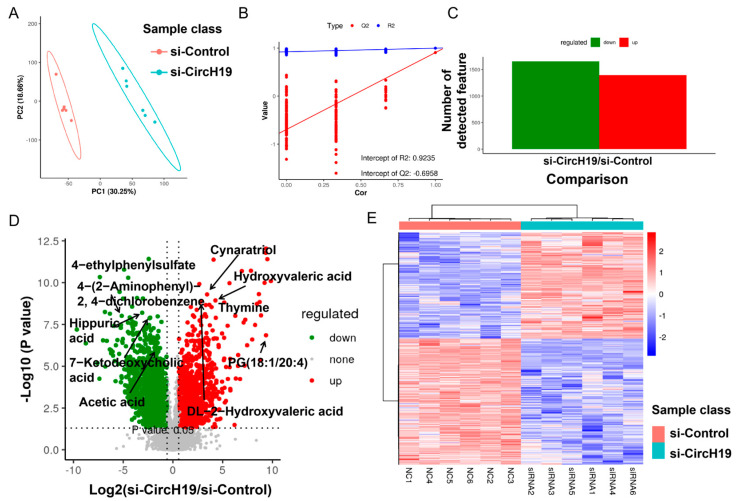
Effect of circH19 knockdown on metabolic profiles. (**A**) Principal component analysis (PCA) plots. (**B**) Permutation tests in the PLS-DA model. (**C**) Bar chart of differential metabolite ions. (**D**) The volcano plot is utilized to display differential metabolites from a univariate statistical test. The plot is designed with the fold-change of the metabolites between comparison groups on the x-axis and the −log10 of the q-value on the y-axis. (**E**) Heatmap of differential metabolites between comparison groups. The expression of differential substances across various samples is depicted in a heatmap after normalizing the intensity values of each metabolite.

Metabolomics techniques were applied to comprehensively assess the consequences of circH19 knockdown on metabolic profiles within adipose tissues, and we employed an array of multivariate statistical models, including PCA and PLS-DA. Principal component analysis (PCA) revealed a clear separation between the control and circH19 knockdown groups, indicating significant metabolic differences (Figure 4A). The PCA score plot showed distinct clustering of samples, with the first principal component (PC1) explaining 30.25% of the variance and the second principal component (PC2) explaining 18.66% of the variance. To further validate the models, permutation tests in the PLS-DA model were conducted, and the PLS-DA analysis yielded substantial R2 and Q2 values of 0.9235 and −0.6958, underscoring the robustness and reliability of our findings (Figure 4B).

Combined with multivariate statistical analysis using PLS-DA, the VIP values were obtained to screen for differentially expressed metabolic ions. A total of 1250 features were quantified, with 564 metabolites upregulated and 686 downregulated in the siCircH19 group compared to the control (Figure 4C). A volcano plot is utilized to display differential metabolites from a univariate statistical test. The plot is designed with the fold-change of the metabolites between comparison groups on the x-axis and the −log10 of the q-value on the y-axis, and volcano plots prominently illustrate the significant impact of circH19 knockdown on the metabolic profiles (Figure 4D). Subsequent to these analyses, we systematically identified the differential metabolites based on fold-changes, *p*-values, and VIP values. We observed that a total of 53 metabolites exhibited upregulation, while 101 metabolites displayed downregulation in circH19 knockdown compared to control mice (as depicted in Supplementary Table S2). To render these alterations more accessible, we employed heatmaps to visually represent the relative intensity of the top 20 identified differential metabolites (Figure 4E, Supplementary Table S2).

### 3.4. Impact of circH19 Knockdown on Metabolic Function and Pathway Analysis in Obese Mice

**Figure 5:**
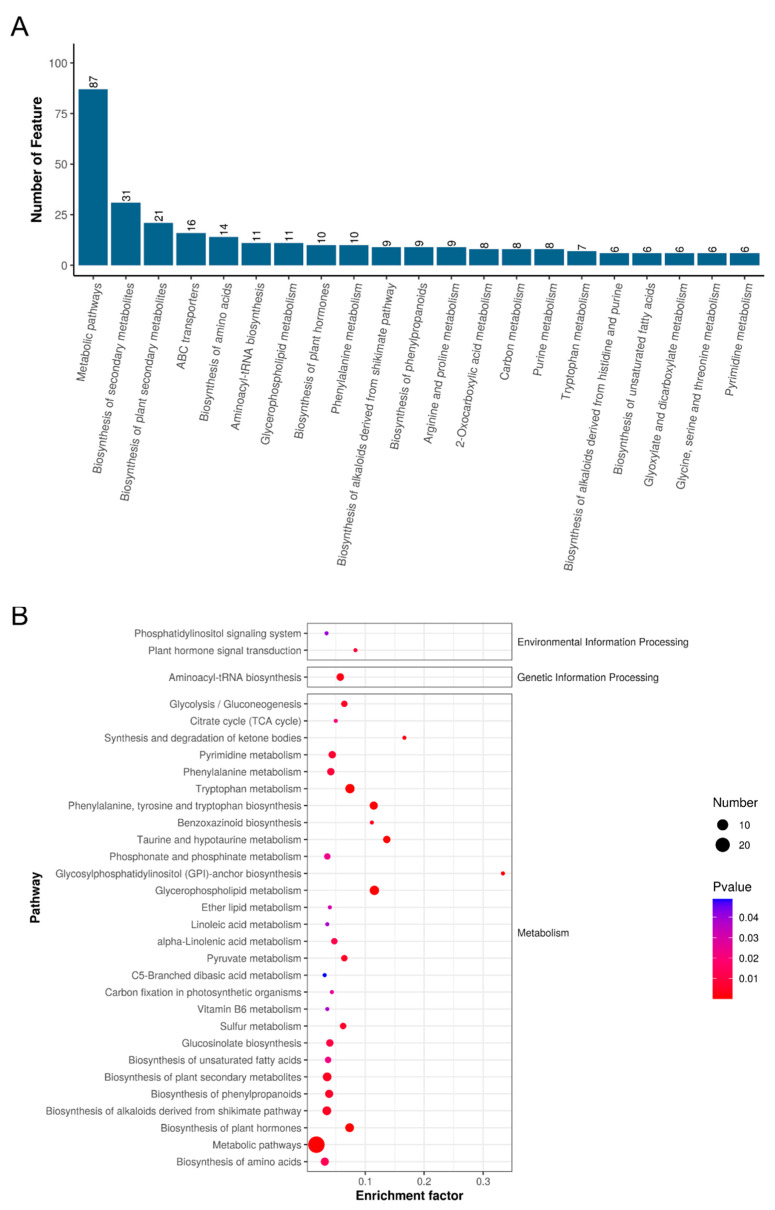
Effect of circH19 knockdown on metabolic pathways. (**A**) Distribution of metabolites across KEGG level 2 categories. The pathway map uses the top 20 pathway entries as the horizontal axis and the number of corresponding metabolites as the vertical axis. (**B**) Enrichment analysis of pathways for differential metabolites utilizing MetaboAnalyst 5.0. Rich Factor is calculated as the ratio of the number of differential metabolites to the total number of metabolites in a given KEGG pathway. The results of the KEGG enrichment analysis are displayed using ggplot2 in the form of a scatter plot, where the Rich Factor indicates the number of differential metabolites in that KEGG pathway divided by the total number of metabolites in the pathway. The smaller the *p*-value, the higher the degree of KEGG enrichment.

The KEGG database was used to explore the metabolic pathways potentially influenced by circH19 knockdown. The figure displays a graphical representation of secondary identified metabolites participating in the pathway distribution across the top 20 of the KEGG classification system. Each bar or segment corresponds to a different category, with the height or length of the bar indicating the number of metabolites identified within that specific category. The maximum number of features is for metabolic pathways, and the second quantity is for the biosynthesis of secondary metabolites (Figure 5A). Pathway analysis of the differential metabolites revealed significant alterations in multiple metabolic pathways, including lipid metabolism, amino acid catabolism, and the tricarboxylic acid (TCA) cycle. The enrichment analysis showed that these pathways were significantly impacted by the circH19 knockdown intervention (Figure 5B, Supplementary Table S3). Our analysis showed that circH19 knockdown significantly affected metabolic pathways, particularly enhancing glycerophospholipid and unsaturated fatty acid biosynthesis, and impacting fatty acid degradation and ketone body metabolism. The upregulation of specific metabolites in these pathways, along with changes in steroidogenesis and amino acid metabolism, suggests circH19’s potential as a therapeutic target for metabolic disorders and lipid regulation.

**Figure 6:**
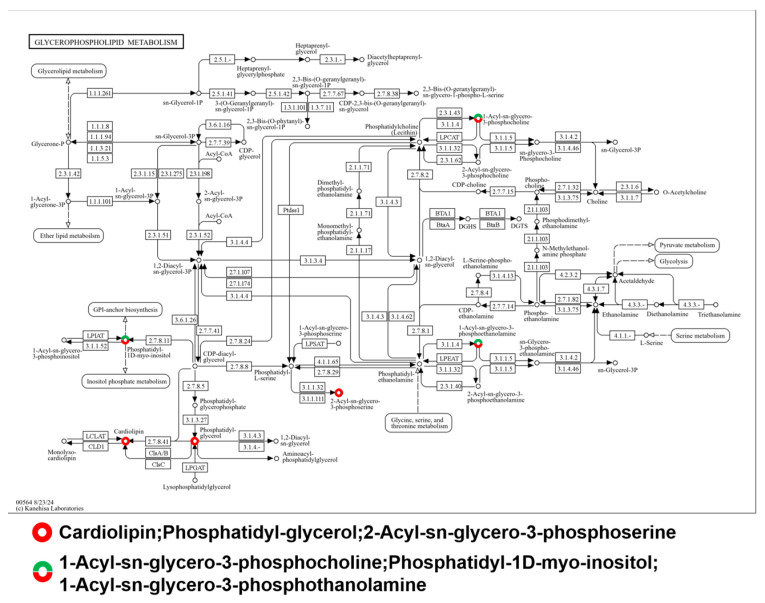
Effect of circH19 knockdown on glycerophospholipid metabolic pathways. A visual representation of glycerophospholipid metabolism pathways. Red metabolites: these represent metabolites that are upregulated or increased in concentration, suggesting that the pathways involving these metabolites are more active under the conditions being studied. Green metabolites: these metabolites are downregulated or decreased in concentration. This indicates that the associated metabolic pathways are less active or that the body or cells are using these metabolites more rapidly than they are being produced.

Taking the glycerophospholipid metabolism pathways as an example, which is a crucial part of lipid metabolism (Figure 6), glycerophospholipids are a class of lipids that play a significant role in the structure of cell membranes and are involved in various cellular processes. Differences in the levels of metabolites can indicate changes in metabolic activity or responses to certain conditions or treatments. The color-coded representation of these metabolites, with red indicating upregulation and green indicating downregulation, offers a visual summary of the metabolic shifts. Specifically, the central metabolite sn-Glycerol-3P, involved in glyceroneogenesis, along with Phosphatidylcholine (PC), a principal membrane component, and Acyl-CoA species, which are pivotal for lipid metabolism, demonstrate the dynamic nature of these pathways. The synthesis of phospholipids, such as PC and PE, facilitated by CDP-choline and CDP-ethanolamine, further underscores the complexity and interconnectivity within the glycerophospholipid metabolic network. These variations in metabolite levels are likely indicative of broader changes in cellular function, energy management, and membrane integrity, which may be responsive to specific physiological or pathological conditions.

## 4. Discussion

Dysregulation of lipid metabolism is emerging as a critical health issue leading to a spectrum of metabolic disorders, such as fatty liver disease, impaired adipose tissue function, diabetes, and obesity. Circular RNAs (circRNAs), characterized by their distinctive structure and broad influence, are a novel class of RNA molecules that have been implicated in a multitude of biological processes, with notable involvement in lipid homeostasis [15,16,17]. Progress in high-throughput technologies has helped clarify the pathophysiology of glycolipid metabolism disorder [18]. The present study investigates the role of circular RNA circH19 in metabolic regulation, particularly in the context of obesity. Our research involved the knockdown of circH19 in a diet-induced obese mouse model and subsequent metabolomic analysis of plasma samples using liquid chromatography–mass spectrometry (LC-MS). Our findings demonstrate that circH19 knockdown leads to significant alterations in plasma metabolites, particularly impacting lipid metabolism and amino acid turnover. These results underscore the potential of circH19 as a regulatory factor in obesity-associated metabolic dysregulation.

In our present study, non-targeted metabolomics serves as a pivotal technique for comprehensively profiling and quantifying the metabolic changes in the plasma of HFD obese mice following circH19 knockdown. Non-targeted metabolomics allows for the unbiased detection and quantification of a wide spectrum of metabolites present in the biological samples, providing a holistic view of the metabolic landscape [19,20]. Due to its untargeted nature, this method is well-suited to discovering novel biomarkers and metabolic pathways that may be affected by circH19 knockdown, which might not have been identified using a targeted approach. The use of liquid chromatography–mass spectrometry (LC-MS) in non-targeted metabolomics ensures high sensitivity and accuracy in metabolite detection, which is crucial for observing subtle changes in the metabolic profile induced by the intervention. The technique is complemented by advanced statistical methods, such as PCA and PLS-DA, thus enabling the identification of differential metabolic signatures and the assessment of their biological relevance. By reflecting the dynamic metabolic status influenced by both endogenous and exogenous factors, non-targeted metabolomics provides insights that are valuable for the diagnosis and monitoring of metabolic disorders, including obesity [2,11]. The plasma metabolome represents an individual’s systemic metabolic status, and non-targeted metabolomics captures this systemic view, which is essential for understanding the complex interactions in obesity-related metabolic dysregulation. In summary, non-targeted metabolomics plays a crucial role in this study by providing a detailed and unbiased metabolic profile that reveals the impact of circH19 on systemic metabolism, identifying potential therapeutic targets, and enhancing our understanding of the metabolic changes associated with obesity.

The observed reduction in circulating lipids following circH19 knockdown is consistent with the hypothesis that circH19 may promote lipid accumulation in obesity. One of our previous studies investigated the role of circH19 in human adipose-derived stem cells (hADSCs) and its association with metabolic syndrome, finding that the knockdown of circH19 promotes adipogenic differentiation and lipid accumulation in hADSCs through a mechanism involving the polypyrimidine tract-binding protein 1 (PTBP1). The research suggests that circH19 may serve as a potential therapeutic target for treating metabolic syndrome by modulating adipose tissue function and lipid metabolism [9]. By modulating the levels or function of circH19, it might be possible to influence adipose tissue function and lipid metabolism, which are critical factors in metabolic health.

The pathway analysis revealed disruptions in energy production pathways; given the critical role of energy metabolism in obesity, the disruptions are noteworthy. The tricarboxylic acid (TCA) cycle, in particular, is a central pathway for the oxidation of nutrients and the generation of ATP. Disruptions in this cycle could lead to reduced energy expenditure and contribute to the development of obesity [21]. The circH19 knockdown intervention appears to activate the TCA cycle, potentially through PPARα upregulation triggered by essential fatty acids, leading to enhanced metabolic efficiency. The activation of PPARα optimizes the TCA cycle’s function, offering a strategy for improving metabolic health and addressing conditions like obesity. Specifically, our analysis revealed the profound impact of circH19 knockdown on various metabolic pathways, with a significant focus on glycerophospholipid metabolism, characterized by the upregulation of metabolites, such as lysophosphatidic acid [22]. Furthermore, the biosynthesis of unsaturated fatty acids demonstrated increased levels of linoleic acid, potentially influencing the overall lipid profile [23]. Ether lipid metabolism also underwent changes, with alterations in the levels of plasmalogens, which are crucial for membrane fluidity and function. In the context of fatty acid degradation, the mitochondrial beta-oxidation of long-chain fatty acids showed a notable upregulation of oleic acid, impacting breakdown and energy production from fatty acids [24].

Additionally, the synthesis and degradation of ketone bodies were affected, with increased levels of beta-hydroxybutyrate, indicating a shift towards ketone body utilization for energy during circH19 knockdown [16]. Steroidogenesis pathways were also influenced, as evidenced by the upregulation of androstenedione, a key intermediate in the production of sex hormones [25]. Moreover, the interconversions of pentose and glucuronate, as well as the metabolism of alpha-linolenic acid, were significantly enriched, reflecting the comprehensive metabolic reprogramming in response to circH19 knockdown. It is noteworthy that the upregulated differential metabolites were particularly enriched in the biosynthesis of unsaturated fatty acids, while the downregulated metabolites were prominently enriched in the metabolism of tryptophan, an essential amino acid. This differential enrichment highlights the complex metabolic repercussions of circH19 knockdown on the obese mice, suggesting that modulating circH19 levels could represent a therapeutic strategy for obesity and related metabolic disorders.

When considering the safety and potential side effects of circH19 knockdown intervention, it is important to address several key points. The safety of any genetic intervention, including circH19 knockdown, depends on the specificity of the method used and the potential off-target effects. By using techniques like RNA interference (RNAi) or CRISPR/Cas9, researchers can aim to minimize off-target effects, but thorough validation is necessary to ensure safety. While circH19 may play a role in disease progression, it could also have essential physiological functions. Knockdown could inadvertently affect these functions, leading to unintended consequences, such as metabolic imbalances, altered gene expression, or impacts on cellular processes. Monitoring subjects’ post-intervention is crucial, including regular health assessments, biochemical profiling, and molecular analysis to identify any deviations from normal physiological parameters that may indicate a side effect. Should side effects be detected, it is important to have protocols in place for intervention management. This could involve adjusting the knockdown strategy, providing supportive treatments, or, if possible, reversing the genetic modification. Given the potential for delayed effects, long-term follow-up is essential to capture any late-onset side effects that may not be immediately apparent. Of course, it is also vital to consider the ethical implications of circH19 knockdown, thus ensuring that the intervention is justified by the potential benefits and that subjects are fully informed of the risks.

This study, while providing valuable insights into the role of circH19 in obesity and metabolic regulation, acknowledges several limitations. Firstly, the research was conducted in a murine model, and the applicability of these findings to human physiology and metabolism requires further investigation. Secondly, the specific molecular mechanisms by which circH19 influences metabolic pathways are not fully elucidated, necessitating more in-depth studies to identify the direct targets of circH19 and its role in cellular metabolic processes. Additionally, while the study identified metabolic changes associated with circH19 knockdown, the long-term effects of modulating circH19 levels on obesity and metabolic health were not assessed and warrant future research. Lastly, the study’s findings may not be broadly generalizable due to the specific conditions and the model used, indicating the need for additional research with diverse populations and experimental setups to confirm the therapeutic potential of circH19 modulation in metabolic disorders.

## 5. Conclusions

In conclusion, our metabolomics study has identified a connection between circH19 and systemic metabolism in HFD obese mice. The circH19 knockdown intervention has the potential to reshape the plasma metabolome, offering a new perspective on the therapeutic modulation of circRNAs in metabolic disorders. Further investigation is needed to fully understand the role of circH19 and to harness its potential in the treatment of obesity.

## Data Availability

Publicly available datasets were analyzed in this study. This data can be found here: https://github.com/linxihua2020/Metabolomics-circH19 (accessed on 17 July 2024).

## Supplementary Materials

The following supporting information can be downloaded at https://www.mdpi.com/article/10.3390/metabo14110603/s1, Supplementary Table S1: Primer sequences for qRT-PCR; Supplementary Table S2: The differential metabolites in circH19-knockdown compared to control mice; Supplementary Table S3: Secondary metabolites identified participate in the pathway distribution across the top 20 of the KEGG classification system.

## References

[1] Lin X., Li H. (2021). Obesity: Epidemiology, Pathophysiology, and Therapeutics. Front. Endocrinol..

[2] Payab M., Tayanloo-Beik A., Falahzadeh K., Mousavi M., Salehi S., Djalalinia S., Ebrahimpur M., Rezaei N., Rezaei-Tavirani M., Larijani B. (2022). Metabolomics prospect of obesity and metabolic syndrome; a systematic review. J. Diabetes Metab. Disord..

[3] Shafiee A., Nakhaee Z., Bahri R.A., Amini M.J., Salehi A., Jafarabady K., Seighali N., Rashidian P., Fathi H., Abianeh F.E. (2024). Global prevalence of obesity and overweight among medical students: A systematic review and meta-analysis. BMC Public Health.

[4] Wang Y., Tang B., Long L., Luo P., Xiang W., Li X., Wang H., Jiang Q., Tan X., Luo S. (2021). Improvement of obesity-associated disorders by a small-molecule drug targeting mitochondria of adipose tissue macrophages. Nat. Commun..

[5] Avelino T.M., Provencio M.G.-A., Peroni L.A., Domingues R.R., Torres F.R., de Oliveira P.S.L., Leme A.F.P., Figueira A.C.M. (2024). Improving obesity research: Unveiling metabolic pathways through a 3D In vitro model of adipocytes using 3T3-L1 cells. PLoS ONE.

[6] Liu C.X., Chen L.L. (2022). Circular RNAs: Characterization, cellular roles, and applications. Cell.

[7] Chen L.L. (2020). The expanding regulatory mechanisms and cellular functions of circular RNAs. Nat. Rev. Mol. Cell Biol..

[8] Glažar P., Papavasileiou P., Rajewsky N. (2014). circBase: A database for circular RNAs. RNA.

[9] Gui W., Zhu W.F., Zhu Y., Tang S., Zheng F., Yin X., Lin X., Li H. (2020). LncRNAH19 improves insulin resistance in skeletal muscle by regulating heterogeneous nuclear ribonucleoprotein A1. Cell Commun. Signal..

[10] Zhu Y., Gui W., Lin X., Li H. (2020). Knock-down of circular RNA H19 induces human adipose-derived stem cells adipogenic differentiation via a mechanism involving the polypyrimidine tract-binding protein 1. Exp. Cell Res..

[11] Ottosson F., Smith E., Ericson U., Brunkwall L., Orho-Melander M., Di Somma S., Antonini P., Nilsson P.M., Fernandez C., Melander O. (2022). Metabolome-Defined Obesity and the Risk of Future Type 2 Diabetes and Mortality. Diabetes Care.

[12] Teruya T., Sunagawa S., Mori A., Masuzaki H., Yanagida M. (2023). Markers for obese and non-obese Type 2 diabetes identified using whole blood metabolomics. Sci. Rep..

[13] Cirulli E.T., Guo L., Swisher C.L., Shah N., Huang L., Napier L.A., Kirkness E.F., Spector T.D., Caskey C.T., Thorens B. (2019). Profound Perturbation of the Metabolome in Obesity Is Associated with Health Risk. Cell Metab..

[14] Ng P., Parks R.J., Cummings D.T., Evelegh C.M., Graham F.L. (2000). An enhanced system for construction of adenoviral vectors by the two-plasmid rescue method. Hum. Gene Ther..

[15] Chen C., Zhang X., Deng Y., Cui Q., Zhu J., Ren H., Liu Y., Hu X., Zuo J., Peng Y. (2021). Regulatory roles of circRNAs in adipogenesis and lipid metabolism: Emerging insights into lipid-related diseases. FEBS J..

[16] Moore M.P., Shryack G., Alessi I., Wieschhaus N., Meers G.M., Johnson S.A., Wheeler A.A., Ibdah J.A., Parks E.J., Rector R.S. (2024). Relationship between serum β-hydroxybutyrate hepatic fatty acid oxidation in individuals with obesity, NAFLD. Am. J. Physiol. Endocrinol. Metab..

[17] Li G., Xu X., Yang L., Cai Y., Sun Y., Guo J., Lin Y., Hu Y., Chen M., Li H. (2023). Exploring the association between circRNA expression and pediatric obesity based on a case-control study and related bioinformatics analysis. BMC Pediatr..

[18] Fang X., Miao R., Wei J., Wu H., Tian J. (2022). Advances in multi-omics study of biomarkers of glycolipid metabolism disorder. Comput. Struct. Biotechnol. J..

[19] López-Yerena A., Domínguez-López I., Vallverdú-Queralt A., Pérez M., Jáuregui O., Escribano-Ferrer E., Lamuela-Raventós R.M. (2021). Metabolomics Technologies for the Identification and Quantification of Dietary Phenolic Compound Metabolites: An Overview. Antioxidants.

[20] Heaney L.M., Deighton K., Suzuki T. (2019). Non-targeted metabolomics in sport and exercise science. J. Sports Sci..

[21] Guasch-Ferré M., Santos J.L., Martínez-González M.A., Clish C.B., Razquin C., Wang D., Liang L., Li J., Dennis C., Corella D. (2020). Glycolysis/gluconeogenesis- and tricarboxylic acid cycle-related metabolites, Mediterranean diet, and type 2 diabetes. Am. J. Clin. Nutr..

[22] van der Veen J.N., Kennelly J.P., Wan S., Vance J.E., Vance D.E., Jacobs R.L. (2017). The critical role of phosphatidylcholine and phosphatidylethanolamine metabolism in health and disease. Biochim. Biophys. Acta Biomembr..

[23] Ravaut G., Légiot A., Bergeron K.F., Mounier C. (2020). Monounsaturated Fatty Acids in Obesity-Related Inflammation. Int. J. Mol. Sci..

[24] Lee J., Choi J., Alpergin E.S.S., Zhao L., Hartung T., Scafidi S., Riddle R.C., Wolfgang M.J. (2017). Loss of Hepatic Mitochondrial Long-Chain Fatty Acid Oxidation Confers Resistance to Diet-Induced Obesity and Glucose Intolerance. Cell Rep..

[25] Huan C., Wang M., Song Y., Jia Z., Wei D., Wang L., Xu Q., Wang J., Zhao M., Geng J. (2024). Inflammatory markers and androstenedione modify the effect of serum testosterone on obesity among men: Findings from a Chinese population. Andrology.

