# Supplementary material for: Profiling of Metabolome in the Plasma Following a circH19 Knockdown Intervention in Diet-Induced Obese Mice
Source: Metabolites. 2024 Nov 8;14(11):603. doi: 10.3390/metabo14110603 (PMC11596236; doi:10.3390/metabo14110603)
Supplement: Supplementary file 1 [file metabolites-14-00603-s001.zip › Supplementary Table S1.pdf]

Supplementary Table S1:
Primer sequences for qRT-PCR.

Gene	Prime
GAPDH	F: 5'-TGAACGGGAAGCTCACTGG-3'
	R: 5'-TCCACCACCCTGTTGCTGTA-3'
CircH19	F: 5'-CCCACAAGCTCTCCTCCA-3′
	R: 5'-GGAGTGTGGAGTAGGCGC-3′
